# Targeted therapy with apatinib in a patient with relapsed small cell lung cancer: A case report and literature review

**DOI:** 10.1097/MD.0000000000009259

**Published:** 2017-12-15

**Authors:** Jun Zhao, Xiaoling Zhang, Chaojie Gong, Jialei Zhang

**Affiliations:** a Department of Oncology, Changzhi People's Hospital, Changzhi, Shanxi; b Department of Geriatrics, Shanghai First People's Hospital, Shanghai Jiao Tong University, Shanghai; c Department of Anesthesiology, Changzhi People's Hospital, Changzhi, Shanxi, China.

**Keywords:** apatinib, small cell lung cancer, targeted therapy, vascular endothelial growth factor receptor

## Abstract

**Rationale:**

Small cell lung cancer (SCLC) is a lethal malignancy. Once relapsed, the disease is irreversible and most of the patients will die of cancer aggravation in 1 to 2 months. In the past several decades, little progress has been made in the systemic treatment of SCLC. Apatinib, as a novel small-molecule tyrosine kinase inhibitor specifically targeting the vascular endothelial growth factor receptor 2 (VEGFR2), has achieved progress in treatment of a variety of cancers. However, there has been no report of the targeted therapy with apatinib in SCLC yet.

**Patient concerns:**

A 63-year-old man, an ex-smoker, presented with a slight hoarseness and cough. The patient was admitted to our department with a primary diagnosis of SCLC at an extensive stage (ES-SCLC). After 17 months of successful first-, second-, and third-line chemotherapy, the disease eventually became relapsed. Then, apatinib treatment started promptly on demand by the patient and his family.

**Intervention:**

After presenting an informed consent, the patient received apatinib treatment immediately at a dose of 250 mg/day orally.

**Outcomes:**

(1) On the 28th day of apatinib therapy, the symptoms of dyspnea and poor appetite of the patient were notably improved. (2) The CT scan taken on the 70th day showed that the pleural effusion in the left lung almost disappeared. (3) The elevated serum neuron-specific enolase (NSE) level was decreased. The patient died of acute respiratory failure on the 172nd day of apatinib treatment. Importantly, the tumor mass did not enlarge obviously during apatinib treatment.

**Lessons:**

Here, we presented a case with relapsed SCLC who unexpectedly responded to single-agent apatinib treatment. Therefore, this report will shed light on future studies of targeted therapy with apatinib in SCLC at different stages.

## 1 Introduction

Small cell lung cancer (SCLC) accounts for 15 to 20% of all thoracic cancers.^[1]^ Its biological characteristics include rapid growing, easy metastasis in early stage and high aggressiveness.^[2]^ Although patients with SCLC are sensitive to the initial chemotherapy, most of them still progress or become relapsed in a few months.^[3]^ Cigarette smoking is critically involved in the carcinogenesis of SCLC and the decreasing prevalence of cigarette smoking has been in parallel with the declining incidence of SCLC in the United States in the past decades.^[4]^ Statistically, a 2-year survival rate is less than 5% for patient with ES-SCLC.^[5]^

In the past several decades, little progress has been made in the systemic treatment of SCLC and the platinum/etoposide-based standard regiment had not met the challenge.^[6]^ A number of clinical trials focused on targeted therapies had not achieved convincing results.^[7]^ Recently, the early phase clinical trials with immune checkpoint inhibitors have been performed. The preliminary data for permbrolizumab and nivolumab, the antibodies blocking the program cell death 1 (PD1), and ipilimumab, the antibody blocking the cytotoxic T-lymphocyte-associated protein 4 (CTLA-4), showed promising anticancer results.^[8]^

Angiogenesis is essential for tumor growth and metastasis, whereas the vascular endothelial growth factor (VEGF) and its receptors (VEGFRs) play a crucial role in angiogenesis.^[9]^ VEGF family includes VEGF-A, VEGF-B, VEGF-C, VEGF-D, and the placental growth factor (PIGF). Three different tyrosine kinase receptors of VEGF are VEGFR1 (Flt-1), VEGFR2 (Flk-1-KDR), and VEGFR3.^[10]^ Upon binding to its receptors, activated VEGF family promotes proliferation of vascular cells to develop new blood vessels in tumor tissues, which, in turn, ensures oxygen and nutrients supply and causes cancer growth and metastasis.^[9,11]^ Among the 3 VEGFRs, VEGFR2 plays a pivotal role in VEGF-mediated cancer angiogenesis.

VEGFR2, as a type II transmembrane tyrosine kinase receptor, can bind VEGF-A, VEGF-C, and VEGF-D. When associated with VEGF-A, the dimerization of VEGFR2 causes autophosphorylation of intracellular tyrosine kinase domains, leading to activation of PLC-γ-Raf kinase-MEK-MAP kinase pathway, which promotes endothelial cell proliferation and survival.^[10]^

Apatinib is a novel antiangiogenic agent specifically targeting VEGFR2.^[12]^ This small molecule tyrosine kinase inhibitor was approved for the second-line treatment of advanced gastric cancer in the People's Republic of China in 2014.^[13]^ It has been currently used in the treatment of a variety of solid tumors, such as advanced gastric cancer, breast cancer, hepatocellular carcinoma, and nonsmall cell lung cancer.^[13]^ In this report, we presented a case with relapsed SCLC who unexpectedly responded to apatinib treatment.

## 2 Case report

A 63-year-old male, an ex-smoker, presented with a slight hoarseness and cough. The CT examination revealed a solitary 3.0-cm pulmonary mass in his left upper lung. The pathological diagnosis of SCLC was made based on CT-guided biopsy. Further examination indicated that tumor metastases existed in the cerebrum and the mediastinal lymph node. The disease was accordingly assessed as ES-SCLC. Among the cancer biomarkers detected in the blood sample of the patient, only NSE level was elevated to 38.02 ng/mL (normal range at 0–15.2 ng/mL). The patient then received 6 cycles of first-line chemotherapy: etoposide 160 mg and cis-platin 40 mg dl-3. The radiotherapy was performed on left upper lung and cerebrum for tumor metastases. The tumor exhibited partially response (PR) to complete response (CR) to both initial chemotherapy and radiotherapy, and the patient experienced an 11-month period of symptomatic improvement time. However, in the following 6 months, the disease grew worse. He had to receive a second-line chemotherapy for 2 cycles: irinotecan 300 mg + carboplatin 500 mg, and a third-line chemotherapy for 2 cycles: lobaplatin 30 mg + cyclophosphamide 900 mg + epirubicin 90 mg. Seventeen months after diagnosis of ES-SCLC, the patient developed clinical symptoms of tiredness, poor appetite, chest distress, shortness of breath, cough and dyspnea, and the tumor-associated hemorrhagic pleural effusion in the left chest was increased. The NSE level was elevated to 61.11 ng/mL. Apparently, the disease became progressive and nonresponsive to standard chemotherapy. Then, the patient agreed to use apatinib as a therapeutic option.

**Figure 1:**
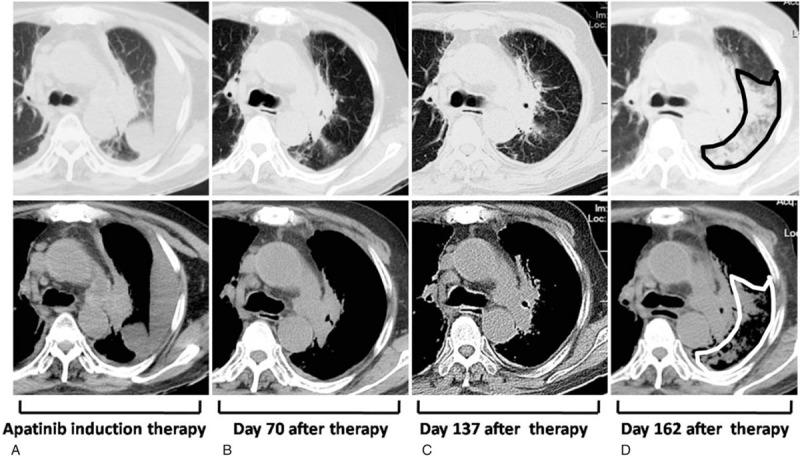
Computed tomograms of the patient after apatinib therapy. (A) Prominent pleural effusion in the left chest of the patient. (B) and (C) the effusion in the left chest almost completely disappeared. (D) Extensive inflammatory infiltration (see the marked area) in the left chest.

After obtaining an informed consent from the patient and the approval of this study from The Ethics Committee of Changzhi People's Hospital, we started apatinib treatment for the patient approximately 17 months after diagnosis of ES-SCLC when all other therapies failed to yield an adequate remission. In view of the poor general condition of our patient and the side effects of apatinib at the routine dosage (500–850 mg/day), we cautiously prescribed a low dose of apatinib at 250 mg/day orally to the patient. To our surprise, on the 28th day of apatinib therapy, the patient's family informed us that the symptoms of the patient were notably improved and asked us to continue the therapy. The CT scan taken on day 70 of the treatment showed that the pleural effusion in the left lung almost completely disappeared (Fig. 1B), as compared with those taken at the beginning of apatinib therapy (Fig. 1A). On the 137th day of the treatment, the similar results of CT scan were achieved (Fig. 1C). During this period of time, the NSE level was decreased from 61.11 ng/mL to 36.28 ng/mL and the side effects of apatinib were minor and tolerable. On the 162nd day of apitinib therapy, the patient was hospitalized due to the pulmonary infection. The CT scan showed a vast inflammatory infiltration in the left lung (Fig. 1D). Ten days later, the patient died of acute respiratory failure. The pulmonary mass was not visibly enlarged in CT images during apatinib treatment.

## 3 Discussion

Despite robust response to initial chemotherapy, the prognosis of patients with ES-SCLC remains very poor. With the rapid development of personalized medicine, the therapeutic regimen for NSCLC has made substantial progress.^[14]^ For example, the specific tyrosine kinase inhibitors (TKIs), gefitinib, erlotinib, and afatinib, which target activating mutations of epidermal growth factor receptor (EGFR), have become first-line standard therapy for EGFR-mutated NSCLC patients.^[15]^ A third-generation TKI, osimertinib, was approved by FDA in 2015 for the treatment of NSCLC patients to overcome acquired resistance to first- and second-generation TKIs.^[16]^ Crizotinib, the anaplastic lymphoma kinase (ALK) inhibitor, has been successfully used in advanced NSCLC with ALK gene rearrangement.^[17]^ Ceritinib and alectinib were recently approved by FDA for the treatment of ALK-positive NSCLC patients who had progressed after crizotinib treatment.^[16]^ For SCLC patients, however, little definitive progress from personalized therapy has been made so far, although the targeted therapies, including antiangiogenic agents, growth factor inhibitors and epigenetic modulators, have been widely studied in SCLC.^[18]^ The clinical trials of immune checkpoint approach via PD1 and CTLA-4 blockage seem to be promising but need further evaluation.^[8]^ Lucchi et al^[19]^ have demonstrated that angiogenesis is critically involved in metastasis of NSCLC and SCLC. However, SCLC has a higher vascularization than NSCLC, as shown by more number of microvessels in the tumor. Therefore, SCLC seems likely to be a better candidate tumor type for the study of new antiangiogenic agents.

In this report, a single-agent therapy with low-dose apatinib remarkably reduced pleural effusion, decreased the elevated serum NSE, and prolonged the survival near 6 months in a relapsed SCLC patient. Since many other antiangiogenic agents have not been shown ideal treatment effects in SCLC, the distinct effect of apatinib observed in this case may not be completely attributed to VEGF/VEGFR-mediated angiogenesis. Besides participating in VEGF activation, VEGFR2 itself possesses strong tyrosine kinase activity.^[10]^ In addition, Mi et al^[20]^ have demonstrated that apatinib reverses the functions of multidrug resistance protein 1 and breast cancer resistance protein through suppressing their transporters. Therefore, the mechanism by which apatinib acts on SCLC is complicated. It should be noted that to test the generalized clinical efficacy of apatinib in treating SCLC a phase II clinical trial entitled: “Apatinib for extensive stage small cell lung cancer after second/third line chemotherapy” was recently launched in China (https://clinicaltrials.gov/ct2/show/NCT02945852).

Overall, herein, we reported a relapsed EX-SCLC patient who had experienced unexpected responsiveness to VEGFR2 inhibitor apatinib. This report will shed light on future studies of apatinib therapeutic strategy in SCLC, including using the agent alone at different stages of the disease, or combination with others.

## Acknowledgments

The authors would like to thank the patient and his family for their agreement on the publication of this report.

## References

[1] Martínez P Sales Fidalgo PA Felip E Ganitumab for the treatment of small-cell lung cancer. Expert Opin Investig Drugs 2014;23:1423–32. 10.1517/13543784.2014.951434 25189625

[2] Lu H Jiang Z Advances in antiangiogenic treatment of small-cell lung cancer. Onco Targets Ther 2017;10:353–9. 28138259 10.2147/OTT.S119714 PMC5238765

[3] Ito T Kudoh S Ichimura T Small cell lung cancer, an epithelial to mesenchymal transition (EMT)-like cancer: significance of inactive Notch signaling and expression of achaete-scute complex homologue 1. Hum Cell 2017;30:1–0. 27785690 10.1007/s13577-016-0149-3

[4] Kalemkerian GP Schneider BJ Advances in small cell lung cancer. Hematol Oncol Clin North Am 2017;31:143–56. 27912830 10.1016/j.hoc.2016.08.005

[5] Slotman BJ van Tinteren H Praag JO Use of thoracic radiotherapy for extensive stage small-cell lung cancer: a phase 3 randomised controlled trial. Lancet 2015;385:36–42. 25230595 10.1016/S0140-6736(14)61085-0

[6] Pietanza MC Byers LA Minna JD Small cell lung cancer: will recent progress lead to improved outcomes? Clin Cancer Res 2015;21:2244–55. 25979931 10.1158/1078-0432.CCR-14-2958 PMC4497796

[7] Stratigos M Matikas A Voutsina A Targeting angiogenesis in small cell lung cancer. Transl Lung Cancer Res 2016;5:389–400. 27652203 10.21037/tlcr.2016.08.04 PMC5009078

[8] Horn L Reck M Spigel DR The future of immunotherapy in the treatment of small cell lung cancer. Oncologist 2016;21:910–21. 27354668 10.1634/theoncologist.2015-0523 PMC4978554

[9] Zhou H Binimadi NO Yang YH Semaphorin 4D cooperates with VEGF to promote angiogenesis and tumor progression. Angiogenesis 2012;15:391–407. 22476930 10.1007/s10456-012-9268-y PMC3733222

[10] Fontanella C Ongaro E Bolzonello S Clinical advances in the development of novel VEGFR2 inhibitors. Ann Transl Med 2014;2:123. 25568876 10.3978/j.issn.2305-5839.2014.08.14 PMC4260048

[11] Kerbel RS Tumor angiogenesis. N Engl J Med 2008;358:2039–49. 18463380 10.1056/NEJMra0706596 PMC4542009

[12] Peng H Zhang Q Li J Apatinib inhibits VEGF signaling and promotes apoptosis in intrahepatic cholangiocarcinoma. Oncotarget 2016;7:17220–9. 26967384 10.18632/oncotarget.7948 PMC4941382

[13] Zhang H Apatinib for molecular target therapy in tumor. Drug Des Devel Ther 2015;13:6075–81. 10.2147/DDDT.S97235 PMC4654530 26622168

[14] Byers LA Rudin CM Small cell lung cancer: where do we go from here? Cancer 2015;121:664–71. 25336398 10.1002/cncr.29098 PMC5497465

[15] Wang X Goldstein D Crowe PJ Next-generation EGFR/HER tyrosine kinase inhibitors for the treatment of patients with non-small-cell lung cancer harboring EGFR mutations: a review of the evidence. Onco Targets Ther 2016;9:5461–73. 27660463 10.2147/OTT.S94745 PMC5021053

[16] Le AD Alzghari SK La-Beck NM Update on targeted therapies for advanced non- small cell lung cancer: nivolumab in contex. Ther Clin Risk Manag 2017;13:223–36. 28260909 10.2147/TCRM.S104343 PMC5328134

[17] Kazandjian D Blumenthal GM Chen H FDA Approval Summary: crizotinib for the treatment of metastatic non-small cell lung cancer with anaplastic lymphoma kinase rearrangements. Oncotarget 2014;19:e5–11. 10.1634/theoncologist.2014-0241 PMC4201002 25170012

[18] Schneider BJ Kalemkerian GP Personalized therapy of small cell lung cancer. Adv Exp Med Biol 2016;890:149–74. 26703804 10.1007/978-3-319-24932-2_9

[19] Lucchi M Mussi A Fontanini G Small cell lung carcinoma (SCLC): the angiogenic phenomenon. Eur J Cardiothorac Surg 2002;21:1105–10. 12048093 10.1016/s1010-7940(02)00112-4

[20] Mi YJ Liang YJ Huang HB Apatinib (YN968D1) reverses multidrug resistance by inhibiting the efflux function of multiple ATP-binding cassette transporters. Cancer Res 2010;70:7981–91. 20876799 10.1158/0008-5472.CAN-10-0111 PMC2969180

